# Does the Integration of Migrants in the Host Society Raise COVID-19 Vaccine Acceptance? Evidence From a Nationwide Survey in Japan

**DOI:** 10.1007/s10903-022-01402-z

**Published:** 2022-09-21

**Authors:** Yuanyuan Teng, Tomoya Hanibuchi, Tomoki Nakaya

**Affiliations:** 1 Center for Northeast Asian Studies, Tohoku University, 41 Kawauchi, Aoba-ku, 980-8576 Sendai, Miyagi Japan; 2 Graduate School of Environmental Studies, Tohoku University, Aoba-468-1 Aramaki, Aoba-ku, 980-0845 Sendai, Miyagi Japan

**Keywords:** Migrants, integration, vaccine hesitancy, COVID-19, Japan

## Abstract

Research indicates that integration contributes to maintaining health among migrants, yet little is known about the association between integration and vaccination acceptance. This study aimed to explore COVID-19 vaccine intention and acceptance, and the association between integration and vaccine hesitancy among migrants in Japan. We conducted an internet survey among migrants in Japan from October 5 to October 14, 2021. Among 1,455 participants, 11.6% reported hesitancy toward COVID-19 vaccination. We found that the overall integration and social integration were associated with the vaccination intention. Some commonly identified barriers (e.g., financial difficulties, language) were not related to COVID-19 vaccination acceptance among migrants in Japan. Highly integrated migrants were less likely to report vaccine hesitancy against COVID-19. To promote COVID-19 acceptance among migrants, customized intervention policies should focus on the migrants with a lower level of integration, especially those with little social connection with the locals.

## Introduction

Vaccination is vital to controlling COVID-19 infection. It is particularly important to promote COVID-19 vaccination among migrants, not only because of the essentiality to achieve global herd immunity, but also because migrants have been one of the most vulnerable groups during the pandemic [1]. Evidence has shown that migrants have a higher risk of exposure to COVID-19 because many of them hold essential jobs and have fewer opportunities to work from home or stay home when they are sick [2, 3]. Further, some migrants also have had difficulties in maintaining physical distancing and self-isolation because of their overcrowded living conditions [2–4]. As a result, higher COVID-19 infection and mortality rates have been observed among migrants and ethnic minority groups [2–6]. Thus, equitable access to COVID-19 vaccines is vital to prevent an increase in social and health inequality among migrants.

Previous studies have shown that, globally, migrants are one of the groups that suffer from under-immunization against vaccine-preventable diseases [7–10]. The factors related to their vaccination behavior include individual-level factors (e.g., place of birth, socioeconomic status, residential duration, personal beliefs or trust, language proficiency) and structural obstacles, such as administrative barriers and health system barriers [11–13]. Furthermore, emerging evidence from European countries shows much greater hesitancy and lower uptake of COVID-19 vaccines among migrants and certain ethnic minority groups [14–18].

However, migrant populations are also diverse, and it is expected that their disadvantage in access to healthcare may decrease in line with their level of integration into the host society. Integration is a concept representing a successful settlement of migrants, which could be measured by the migrants’ knowledge and capacity to build a successful, fulfilling life in the host society [19–21]. Previous studies have indicated that integration contributes to maintaining health among migrants, and poor integration increases health vulnerabilities [22–25]. Research has shown that migrants living in a society with problematic integration policies are more likely to report poor health; furthermore, barriers to socioeconomic integration and discriminatory processes may lead to a higher risk of depression among migrants [23, 24]. Regarding vaccine acceptance, a study in China found that migrants’ integration (e.g., employment, language, and discrimination perception) was related to the complete vaccination of their children [22].

Integration includes multiple aspects, such as psychological, social, economic, political, navigational, and linguistic dimensions [21]. Psychological integration refers to an emotional bond with the host society, while social integration refers to social contacts in the host country [26]. As collective responsibility is a vital factor for vaccination intention [27], a stronger sense of belonging to the host country and increased contact with locals may enhance vaccination motivation. Economic integration is one of the most important aspects of integration; more economic resources can help mitigate constraints (e.g., unpaid leave and financial burden) affecting vaccination behaviors. In this study, political and navigational integration refer to basic knowledge for navigating the host country’s political system and social institutions [21]. Lack of political or navigational integration can reinforce structural obstacles to vaccination. Furthermore, language is a commonly recognized barrier for migrants’ access to healthcare services in the host country; therefore, limited linguistic integration could be a major constraint in vaccination. Thus, the overall integration of migrants may facilitate their COVID-19 vaccine intentions. However, the association between integration and vaccination acceptance among migrants has not yet been examined.

COVID-19 vaccination in Japan began on February 17, 2021, as a staged roll-out process according to priority groups; it prioritized medical staff, followed by older adults and people with chronic illnesses [28]. COVID-19 vaccines are free for all residents of Japan, including migrants, regardless of citizenship status or migration background. As of October 4, 2021, the proportion of the population who had received the first and second doses was 67.2% and 58.2%, respectively [29]. Many studies have been conducted to explore the determinate of vaccine intention among Japanese: women, young people, single people, urbanites, people with low socioeconomic status, and those with severe psychological distress were more likely to be hesitant to accept the COVID-19 vaccine [30–34]. However, little is known about the attitudes toward COVID-19 vaccination among migrants in Japan, despite a growing foreign population. To fill this gap, this study aimed to explore migrants’ intention to receive the COVID-19 vaccine and their reasons for hesitancy, as well as the association between integration and vaccine acceptance.

## Method

### Participants and Data Collection

The Japanese government has enacted a series of policies to attract foreign populations (e.g., international students; migrant workers, both skilled and unskilled) since the 1990s in response to the lack of labor due to the aging Japanese population. In 2019, the foreign population in Japan reached a record 2.9million, comprising 2.3% of the whole population [35]. Most of the foreign population in Japan were from Asian countries (84.1%; mainly from China: 26.8%, Vietnam: 15.4%, South Korea: 14.6%; Philippines: 9.7%), while the proportions of residents from Europe, Africa, North America, South America, and Oceania were 2.7%, 0.7%, 2.6%, 9.3%, and 0.5%, respectively [36]. All migrants with a registered residence have the same access to national health care services as Japanese citizens.

We conducted a cross-sectional survey on COVID-19 and integration among migrants in Japan from October 5 to October 14, 2021. The questionnaire was available in Japanese, English, and plain Japanese. The questions developed in Japanese were translated into English, and vice versa, by bilingual speakers on the research team. Further, in consideration of those who do not quite understand either Japanese or English, the questionnaire was also available in plain Japanese (*Yasashii Nihongo*, a writing style using easy phrases and fewer Chinese characters) edited by a language editing company (Dank Co., Ltd.; https://www.dank-yasanichi.jp/). In a pilot survey, we tested the questionnaire with two members from foreigner support groups and 14 migrants with various language capacities and backgrounds from nine different countries. We revised the questionnaire by replacing difficult words, highlighting easily overlooked information, and providing further instructions to improve understandability and translation accuracy. We conducted the survey online through a survey company, GMO Research (https://gmo-research.com/), which has the largest online survey panel network in Japan. As of October 2021, the network can access 23.6million residents in Japan (https://gmo-research.jp/service/panel/jcp), including over 20,000 foreign nationals. We invited all active registrants of foreign nations who are over 20 years old, regardless of the COVID-19 vaccination status and intention, to participate in our survey. In total, 2,258 participants from 52 countries and regions completed the questionnaire.

**Fig. 1:**
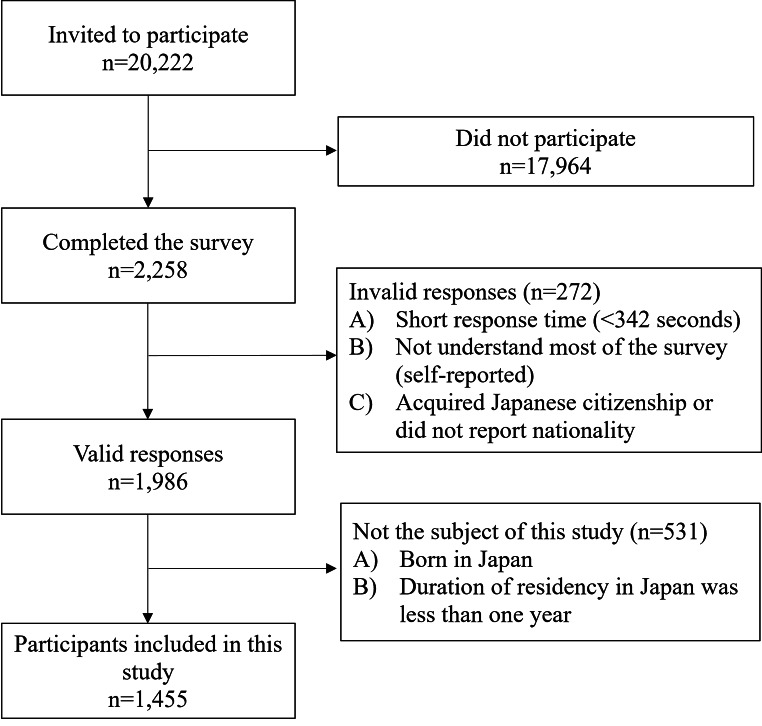
Flow chart of the data collection

To ensure data quality, we adopted a response time approach and self-reported diligence approach to detect careless or insufficient effort in responding [37–39]. Based on a recommended cutoff of 2s per item [38], we excluded responses that took less than 342s for completion. In addition, we discarded the answers from those who reported that they did not understand most of the survey through a self-reported item on understanding at the end of the survey. Further, because our focus was on foreign-born migrants who had been living in Japan before the COVID-19 pandemic, the responses from locally-born second-generation immigrants and those whose duration of residency in Japan was less than one year were excluded from the study. Consequently, the responses of 1,455 participants from 48 countries and regions were used in the analysis (Fig.1). The proportions of participants who answered the questionnaire in Japanese (*n* = 1,217), plain Japanese (*n* = 94), and English (*n* = 144) were 83.7%, 6.5%, and 9.9%, respectively.

This study design was approved by the ethics review board of the Center for Northeast Asian Studies of Tohoku University (CNEAS-ER2021-04). Informed consent was obtained from all participants.

### Measures

#### Vaccine Intention

Vaccine hesitancy was defined as a delay in acceptance or refusal of vaccination despite the availability of vaccination services [40]. We first asked all participants about their COVID-19 vaccination experience. Those who were not fully vaccinated were asked about their intention to receive the vaccine through the question “Do you plan to get vaccinated?” with the possible answers “Yes, I have already made an appointment,” “Yes, but I haven’t made the appointment,” “No, I don’t want to be vaccinated,” and “I haven’t decided yet.” We defined those who reported that they do not want to be vaccinated or have not decided as the hesitant group. The rest, those who were already fully vaccinated or planned to be vaccinated, were defined as the intent group.

**Fig. 2:**
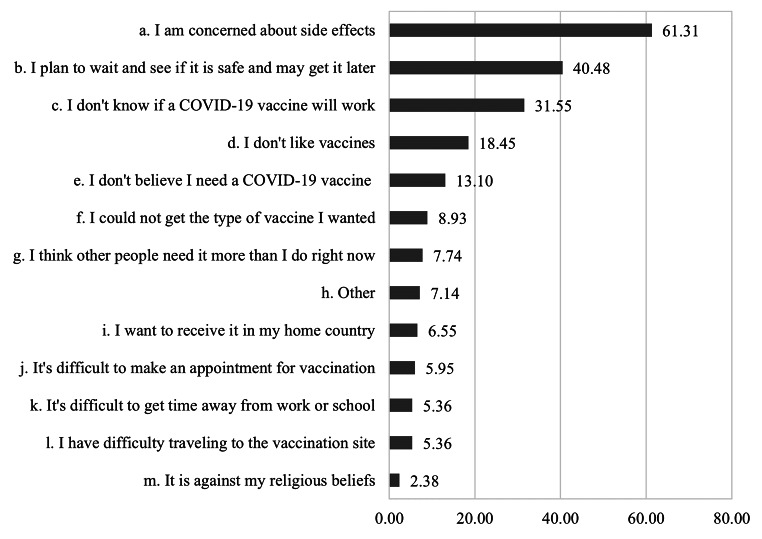
Reasons for hesitancy toward COVID-19 vaccination (n = 168)

Furthermore, we inquired about the reason for their vaccine hesitancy using a multiple-choice question with 12 response options (e.g., “I am concerned about the side effects,” “It’s difficult to get time away from work or school”) (Fig.2). These options were mainly related to how participants think and feel about COVID-19 vaccines (a, b, c, d, e, i, and g) and their barriers to vaccine access (f, j, k, l, and m) [41]. Ten options were adopted from the COVID-19 symptom survey (Version 11, 06/07/2021-07/23/2021; https://covidmap.umd.edu/), based on Japan’s vaccination situation. For example, as the COVID-19 vaccination is free in Japan, we did not include the option “I am concerned about the cost of a COVID-19 vaccine.” Two response options (i.e., “I want to receive it in my home country,” and “It’s difficult to make an appointment for vaccination”) were added based on the pilot test.

#### Integration

We adopted the short form of the Immigration Policy Lab Integration Index (IPL-12) to measure the integration, which was designed to be universal for meaningful comparisons across all migrant groups [21]. The scale captures six critical dimensions of integration—psychological, economic, political, social, linguistic, and navigational—with two survey items for each (Table1). We revised the item measuring social integration to ask participants for the frequency with which they had dinner with Japanese who were not part of their family before the outbreak of the COVID-19 pandemic instead of the frequency of the previous 12 months used in the original scale. Because in-person interactions have changed tremendously as a result of social distancing measures during the past year and may differ according to the infection situation of residential areas, the amount of in-person interaction with locals before the pandemic may better reflect the social integration of migrants.

**Table 1:** Measurement of integration

	Measurement questions	Values	n	Mean	S.D.
Psychological integration	*How connected do you feel with Japan?	do not feel a connection at all = 1; weak = 2; moderately close = 3; very close = 4; extremely close = 5.	1,455	3.63	1.01
	*How often do you feel like an outsider in Japan?	always = 1; often = 2; sometimes = 3; rarely = 4; never = 5.	1,455	3.27	0.96
Linguistic integration	*Your own skills in Japanese: I can **read** and understand the main points in simple newspaper articles on familiar subjects.	not well at all = 1; not well = 2; moderately well = 3; well = 4; very well = 5.	1,455	4.16	1.07
	*Your own skills in Japanese: In a conversation, I can **speak** about familiar topics and express personal opinions.	not well at all = 1; not well = 2; moderately well = 3; well = 4; very well = 5.	1,455	4.23	0.94
Economic integration	Equivalized income (million yen) was calculated according to participants’ annual household income and household size.	less than 1.03 = 1; 1.04 to 2.06 = 2; 2.07 to 3.09 = 3; 3.10 to 4.12 = 4; 4.13 or more = 5.	1,204	3.54	1.42
	What you have been doing for the last 4 weeks?	unemployed = 1; in school/doing unpaid housework/retired = 3; in paid work = 5.	1,453	4.33	1.21
Political integration	*How well do you understand the important political issues facing Japan?	not well at all = 1; not well = 2; moderately well = 3; well = 4; very well = 5.	1,455	3.29	0.99
	In the last 12 months, how often did you typically discuss major political issues facing Japan with others?	never = 1; once a year = 2; once a month = 3; once a week = 4; almost every day = 5.	1,455	2.45	1.24
Social integration	Before the outbreak of the COVID-19 pandemic, how often did you eat dinner with Japanese who are not part of your family?	never = 1; once a year = 2; once a month = 3; once a week = 4; almost every day = 5.	1,455	2.67	1.22
	With how many of them did you have a conversation - either by phone, messenger chat, or text exchange - in the last 4 weeks?	0 = 1; 1 to 2 = 2; 3 to 6 = 3; 7 to 14 = 4; 15 or more = 5.	1,455	2.76	1.28
Navigational integration	In Japan, how difficult or easy would it be for you to see a doctor?	very difficult = 1; somewhat difficult = 2; neither difficult, nor easy = 3; somewhat easy = 4; very easy = 5.	1,455	3.75	1.28
	In Japan, how difficult or easy would it be for you to search for a job (find proper listings)?	very difficult = 1; somewhat difficult = 2; neither difficult, nor easy = 3; somewhat easy = 4; very easy = 5.	1,455	3.24	1.32
* Reverse coded.					

The answers for each question were assigned a value from 1 to 5. The values were summed to form a score for each dimension. The scores for overall integration were the sum score of six dimensions, which ranged from 12 to 60. Higher scores indicate better integration. In the analysis, we applied min-max normalization to rescale scores for overall integration and integration of each dimension from 0 to 1.

#### Control Variables

The control variables included gender, age, marital status, education level, duration of total years in Japan, income level of the home country, residential region, and COVID-19 infection experience. As household income is one of the items measuring economic and overall integration, we chose to control for income level of the migrants’ home country rather than their household income. The income level of the home country was coded according to the classification of the World Bank [42]. Because there were only two participants from low-income countries, we merged the groups of low income and lower-middle income. Residential regions of participants were divided into metropolitan areas and non-metropolitan areas. Metropolitan areas included 11 regions: Saitama, Chiba, Tokyo, Kanagawa, Gifu, Aichi, Mie, Kyoto, Osaka, Hyogo, and Nara Prefectures. The rest of the regions were coded as non-metropolitan areas.

### Analysis

We first calculated descriptive statistics for all variables among full samples and by vaccine hesitancy status. All background variables, overall integration, and integrations of six dimensions were compared between intent and hesitant groups using chi-square tests for categorical variables and *t*-tests for continuous variables.

A multiple imputation approach was adopted to deal with the missing values in economic integration resulting from unreported household income or working status. Twenty imputed data sets were generated. Variables that were incorporated in the multiple imputation procedure included all variables used in the analysis. After imputed data sets were generated, scores for overall integration were calculated.

Following this, we performed multiple logistic regressions to examine the association of vaccine intention with rescaled integration scores to identify which dimension of integrations is related to vaccine hesitancy. Statistical analyses were performed using Stata/SE 17.0.

## Results

### Sample Description

The sociodemographic characteristics of the participants included in this study (*n* = 1,455) by vaccine intention are described in Table2. The mean age of participants was 37.3 years, of whom 61.4% were female.

**Table 2:** Characteristics of the participants and prevalence of hesitancy toward COVID-19 vaccination

	Full sample		Intent / fully vaccinated group (n = 1287)	Hesitant group (n = 168)	
	n / Mean	% / SD	% / Mean	% / Mean	*p*
total	1,455	100.00	88.45	11.55	
Gender					0.970
Female	894	61.44	88.48	11.52	
Male	561	38.56	88.41	11.59	
Age					0.668
20–29	404	27.77	87.13	12.87	
30–39	519	35.67	88.82	11.18	
40–49	313	21.51	88.18	11.82	
50–59	158	10.86	89.24	10.76	
≥ 60	61	4.19	93.44	6.56	
Marital status					0.131
Single/divorced/widowed	674	46.32	87.09	12.91	
Married	781	53.68	89.63	10.37	
Educational level					<0.001***
High school or below	282	19.38	81.21	18.79	
Vocational school/college	236	16.22	88.14	11.86	
University or above	937	64.40	90.72	9.28	
Duration of total years in Japan					0.036*
< 3 years	137	9.42	92.70	7.30	
3–10 years	484	33.26	90.70	9.30	
10–20 years	383	26.32	85.64	14.36	
≥ 20 years	451	31.00	87.14	12.86	
Income level of home country					0.065
Low or lower-middle income	197	13.54	89.85	10.15	
Upper-middle income	606	41.65	86.14	13.86	
High income	652	44.81	90.18	9.82	
Residential region					0.581
Non-metropolitan areas	314	21.58	87.58	12.42	
Metropolitan areas	1,141	78.42	88.69	11.31	
Infected with the COVID-19					0.177
No/don’t know	1,359	93.40	88.15	11.85	
Yes	96	6.60	92.71	7.29	
Overall integration	0.58	0.17	0.55	0.58	0.022*
Psychological integration	0.61	0.21	0.59	0.61	0.215
Linguistic integration	0.80	0.23	0.78	0.80	0.231
Economic integration	0.74	0.26	0.71	0.75	0.103
Political integration	0.47	0.22	0.43	0.47	0.024*
Social integration	0.43	0.25	0.39	0.43	0.058
Navigational integration	0.62	0.29	0.62	0.62	0.872

SD: standard deviation.

Note. Statistically significant differences are highlighted in bold. *p< .05, ***p< .001.

### Descriptive Statistics

We found that among 1,455 participants, 66.3% (*n* = 964) had already been fully vaccinated, and 22.2% had received the first dose and planned to receive the second dose. About 12% of our sample (11.6%) showed hesitancy toward the COVID-19 vaccine (do not want to be vaccinated: 6.7%; have not decided yet: 4.9%). Figure2 displays the reasons for hesitancy. Among respondents who hesitated to be vaccinated (*n* = 168), the top reason given was concerns about side effects (61.3%), followed by concerns about the vaccine’s safety (40.5%) and efficacy (31.6%).

Regarding the relationship between integration and vaccine hesitancy, the political integration score (*p* = .024) and the overall integration (*p* = .002) in the hesitant group were lower than in the intent group.

In addition, significant differences in vaccine intention were noted by educational level, living duration in Japan, and income level of the migrant’s home country. The percentages of vaccine hesitancy were relatively high among those whose educational level was high school or below (18.8%), those who had been living in Japan for more than ten years (10–20 years: 14.4%, more than 20 years: 12.9%), and those who were from upper-middle-income countries (13.9%).

### Multivariable Logistic Regression

Table3 presents the results from multivariable logistic regression analyses examining the relationship between COVID-19 vaccine hesitancy and integration. Overall integration was negatively associated with vaccine hesitancy. Moreover, a significant association was only found between vaccine intention and social integration of the six dimensions. Further, although migrants with a higher level of political integration were more likely to accept the COVID-19 vaccine, political integration was not the determinant factor of the vaccine intention after adjusting for socioeconomic characteristics and other dimensions of integration.

**Table 3:** Logistic regression models for odds ratio of COVID-19 vaccine hesitancy

	Crude OR		95% CI		Overall integration				Dimensions of integration			
					Adjusted OR		95% CI		Adjusted OR		95% CI	
Gender												
Female	0.99		0.71	1.38	0.84		0.59	1.19	0.83		0.58	1.17
Male	1.00 (reference)				1.00 (reference)				1.00 (reference)			
Age												
20–29	1.00 (reference)				1.00 (reference)				1.00 (reference)			
30–39	0.85		0.57	1.27	0.79		0.51	1.23	0.75		0.48	1.18
40–49	0.91		0.58	1.42	0.64		0.37	1.10	0.60		0.34	1.06
50–59	0.82		0.46	1.46	0.53		0.26	1.07	0.50		0.25	1.03
≥ 60	0.48		0.17	1.36	0.29	*	0.09	0.92	0.28	*	0.09	0.89
Marital status												
Married	0.78		0.57	1.08	0.72		0.50	1.03	0.69		0.48	1.00
Single/divorced/widowed	1.00 (reference)				1.00 (reference)				1.00 (reference)			
Educational level												
High school or below	2.26	***	1.56	3.28	1.83	**	1.21	2.76	1.90	**	1.24	2.91
Vocational school/college	1.32		0.84	2.07	1.19		0.75	1.91	1.23		0.76	1.98
University or above	1.00 (reference)				1.00 (reference)				1.00 (reference)			
Duration of total years in Japan												
< 3 years	0.53		0.26	1.07	0.32	**	0.14	0.75	0.34	*	0.14	0.79
3–10 years	0.69		0.46	1.05	0.49	*	0.28	0.85	0.48	*	0.27	0.85
10–20 years	1.14		0.76	1.69	0.96		0.61	1.51	0.97		0.62	1.54
≥ 20 years	1.00 (reference)				1.00 (reference)				1.00 (reference)			
Income level of home country												
Low or lower-middle income	1.04		0.61	1.76	0.76		0.43	1.34	0.77		0.43	1.37
Upper-middle income	1.48	*	1.05	2.09	1.12		0.77	1.63	1.11		0.76	1.62
High income	1.00 (reference)				1.00 (reference)				1.00 (reference)			
Residential region												
Metropolitan areas	0.90		0.61	1.32	1.02		0.69	1.52	1.02		0.68	1.52
Non-metropolitan areas	1.00 (reference)				1.00 (reference)				1.00 (reference)			
Infected with the COVID-19												
Yes	0.59		0.27	1.29	0.59		0.26	1.34	0.61		0.27	1.38
No/don’t know	1.00 (reference)				1.00 (reference)				1.00 (reference)			
Overall integration	0.32	*	0.12	0.83	0.18	**	0.06	0.53				
Psychological integration	0.61		0.28	1.33					0.59		0.26	1.33
Linguistic integration	0.67		0.34	1.29					0.63		0.28	1.39
Economic integration	0.62		0.32	1.18					0.94		0.43	2.04
Political integration	0.43	*	0.21	0.89					0.68		0.31	1.52
Social integration	0.54		0.28	1.02					0.45	*	0.22	0.93
Navigational integration	0.96		0.55	1.65					0.96		0.50	1.85

***p < .001, **p < .01, *p < .05. OR: odds ratio. CI: confidence interval.

Note. Consistent findings were observed by comparing results from the analyses with imputed data and the results from the analyses with complete data.

## Discussion

This is the first attempt to explore vaccine intention among migrants in Japan and the relationship between integration and vaccine hesitancy. We found that 11.6% of migrants were hesitant to be vaccinated against COVID-19. Although it is difficult to compare this figure with other studies as vaccination intentions change over time, vaccine intention among migrants was still not lower than that among Japanese locals. A study found that 11.3% of Japanese citizens were hesitant to be vaccinated before the availability of the COVID-19 vaccines [33]. Another study conducted after the vaccine roll-out found that only 72.4% of Japanese citizens intended to get vaccinated against COVID-19 [34]. However, migrants’ vaccine hesitancy has been found to differ by host society; the proportion of vaccine hesitancy among migrants in Japan was much lower compared with migrants in other settings. For example, 25% of temporary foreign workers from Bangladesh reported vaccine hesitancy [43], 42.3% of people with migratory backgrounds in Munich had considered getting vaccinated [16], and only 41.2% of undocumented migrants in western countries reported they would get the COVID-19 vaccine [44].

The main reason for hesitancy toward COVID-19 vaccination among our participants was a lack of confidence in the vaccine, such as concerns about vaccine harm (e.g., side effects and safety) and its benefits (e.g., effectiveness). This result is similar to the reasons among the Japanese population [32] and is also consistent with previous studies showing that low confidence in vaccine effectiveness and concern about safety correlate with not getting vaccinated [41], which indicates that interventions to increase confidence are vital for both locals and migrants. Furthermore, although we found that barriers to vaccine access were not the primary reason for hesitancy, reducing these barriers may facilitate vaccination to some degree [41]. As the most commonly reported barrier-related reason was “I could not get the type of vaccine I wanted,” increasing the freedom to choose their preferred vaccine could reduce such barriers.

We also found that the vaccine intention was associated with overall integration (*p* = .002). Those with better knowledge and capacity to build a successful life in the host society were less likely to report vaccine hesitancy against COVID-19. Because integration is a complex and bidirectional process, the receiving society’s acceptance also plays an important role during this process [45, 46]. Efforts and policies at both community and national levels for migrant integration may reduce migrants’ hesitancy toward COVID-19 vaccination, thus reducing health disparities.

Among the six dimensions of integration, migrant’s vaccine intention was found to be related to social integration. Vaccination acceptance was higher among migrants who had more interaction with locals and vice versa (*p* = .031). This result is consistent with previous studies indicating that social integration could benefit health [24, 25, 47, 48]. Increased contact with the local community could improve migrants’ access to information regarding the COVID-19 vaccine and boost their trust toward the host society and its medical system, thus reducing concerns about vaccine safety and efficacy and increasing vaccine uptake. Further, because protective motivation is an important psychological determinant of vaccination acceptance [31, 34], more contact with the host community may promote willingness to protect others by being vaccinated. This result also suggests a need to promote vaccination among those who have little connection with locals. Intervention in collaboration with the migrant community may be effective, because those migrants may be closer to their own community. Further, because integrated individuals may be subject to social controls and the attitude among locals may affect the migrant’s intention [41, 47], mitigating the hesitancy among Japanese is also crucial in facilitating vaccination among migrants.

Although language has been one of the dominant barriers to access to health services faced by migrants [7, 10], we did not find a significant relationship between vaccine intention and linguistic integration. This may be because most of our participants lived in metropolitan areas (78.4%), where multilingual information and services are relatively well provided for the concentrated foreign population. Additionally, because the questionnaire was in Japanese and English, the participants in this study may have relatively high language skills enabling them to acquire information in Japan. As shown in a study on the difficulties experienced by Brazilian migrants in Japan, language barriers may be more severe for those who can only speak a minority language and live in a region with few foreigners [49].

We also did not find a significant association between the COVID-19 vaccine intention and economic integration, a commonly identified barrier to vaccination. Access to COVID-19 vaccines is free for all residents, whereas vaccines for other diseases are at the expense of patients who need health insurance to claim a subsidy. As highlighted by Brewer et al. (2017), interventions such as reducing barriers can directly facilitate vaccination uptake [41]; our work adds to existing evidence that free access to vaccines may effectively promote vaccination among groups that are under-immunized due to financial burden. Additionally, migrants with higher education and older adults (≥ 60 years) were less likely to be hesitant; this result was also found among Japanese citizens [30, 50]. Migrants with lower educational levels may lack knowledge regarding the COVID-19 vaccine, leading to a lower vaccination intention [51]. As Japan is a severely aging society and older adults are at higher risk of contracting COVID-19, the Japanese government has implemented policies targeting this group (e.g., priority vaccination); this may have raised vaccine uptake among older adults in both local and migrant groups [50].

The total number of years spent in Japan was related to vaccine hesitancy only after controlling for sociodemographic characteristics and integration levels, indicating that migrants who had lived in Japan for a long time and had a higher level of integration were less likely to exhibit vaccine hesitancy. Newcomers also tended to report lower vaccine hesitancy because they have relatively higher mobility and required COVID-19 vaccination for traveling across the border, which may have increased their vaccination intention. Tailored interventions for those with low educational level individuals, the younger generation, and those with relatively long duration of stay in Japan but a low integration level should be designed as part of vaccination promotions among migrants.

Although our study addresses important gaps in the knowledge on COVID-19 vaccination acceptance and integration among migrants, a few limitations should be mentioned. First, we did not use probability sampling because random sampling that targeted the foreign population was not virtually feasible owing to the unavailability of a complete sampling frame as well as considerable time and financial constraints. In fact, the lack of data on migrants has been a long-standing problem, which was highlighted even more during the COVID-19 pandemic [2, 3, 52, 53]. However, we attempted to reach as many migrants as possible by conducting the survey using the largest online survey panelist network (approximately 19.2% of the population in Japan). Because online survey monitors tend to be more educated and have better socioeconomic status [54], the degree of integration among participants in this study may be higher than that of other migrants. Thus, associations between integration and vaccine intention could be less clear and underestimated in the current study.

Second, though translation and back-translation is a desired practice in multilingual surveys, we did not implement this method as the English version of the questionnaire was an additional option. As we expected most participants to answer the questionnaire in Japanese as they are monitors for surveys usually conducted in Japanese, we focused on improving the comprehension of the Japanese questionnaire. Nevertheless, through the pilot test, we made efforts to ensure that the questions in different languages reproduced the same meaning. Additionally, we found that social integration was significantly related to vaccine hesitancy, but it should be noted that one of the items measuring social integration was revised to reflect social contact before the pandemic.

Furthermore, the definition of vaccine hesitancy is heavily debated in the literature [41]. A strict differentiation between beliefs and behaviors may be more desirable, as, for example, those who were hesitant at the time of the survey may get vaccinated afterward. Nevertheless, we adopted the definition that mainly focuses on vaccine behavior, as the COVID-19 vaccine campaign was already underway during our survey period, and it was impossible to strictly distinguish the respondents’ motivational state from their actual behavior. Last, because vaccination intention differed according to the time, region, and population, our findings may be limited to migrants in Japan during the period when the government was promoting the vaccination energetically. Thus, further studies across time frames and among migrants whose integration is relatively low are required.

## Conclusion

Overall, integration and social integration were associated with the vaccination intention among migrants in Japan. Some commonly identified barriers (e.g., financial difficulties, language) were not related to COVID-19 vaccination acceptance among migrants in Japan. To promote COVID-19 acceptance among migrants, customized intervention policies should focus on migrants with a lower level of integration, especially those with little social connection with locals.
